# Comparison of the immune response during acute and chronic *Staphylococcus aureus* infection

**DOI:** 10.1371/journal.pone.0195342

**Published:** 2018-03-29

**Authors:** Rebecca A. Brady, Christopher P. Mocca, Roger D. Plaut, Kazuyo Takeda, Drusilla L. Burns

**Affiliations:** 1 Division of Bacterial, Parasitic, and Allergenic Products, Center for Biologics Evaluation and Research, FDA, Silver Spring, Maryland, United States of America; 2 Microscopy and Imaging Core Facility, Center for Biologics Evaluation and Research, FDA, Silver Spring, Maryland, United States of America; ContraFect Corp, UNITED STATES

## Abstract

*Staphylococcus aureus* bacteria are able to grow in a planktonic state that is associated with acute infections and in biofilms that are associated with chronic infections. Acute infections, such as skin infections, are often self-limiting. However, chronic infections, such as implant infections, can be difficult to clear and may require surgical intervention. The host immune response may contribute to the different outcomes often associated with these two disease types. We used proteomic arrays and two murine models for an initial, descriptive characterization of the contribution of the host immune response to outcomes of acute versus chronic *S*. *aureus* disease. We compared the immune responses between a model of self-limiting skin and soft tissue infection caused by the planktonic form of *S*. *aureus* versus a model of surgical mesh implant infection, which we show to be caused by a bacterial biofilm. The significantly altered host cytokines and chemokines were largely different in the two models, with responses diminished by 21 days post-implantation in surgical mesh infection. Because bacterial levels remained constant during the 21 days that the surgical mesh infection was followed, those cytokines that are significantly increased during chronic infection are not likely effective in eradicating biofilm. Comparison of the levels of cytokines and chemokines in acute versus chronic *S*. *aureus* infection can provide a starting point for evaluation of the role of specific immune factors that are present in one disease manifestation but not the other.

## Introduction

*Staphylococcus aureus* is a leading cause of both nosocomial and community-acquired infections in the United States [1, 2]. *S*. *aureus* is capable of colonizing and damaging the host while evading host immune responses due to its extensive and redundant repertoire of virulence factors. *S*. *aureus* isolates are often resistant to one or more classes of antibiotics, with the most common resistant strains belonging to the methicillin resistant *S*. *aureus* (MRSA) classification. The ability to acquire resistance and adapt to survive in the face of currently available therapies highlights the importance of vaccine development that would help prevent *S*. *aureus* infection occurrence.

*S*. *aureus* is proficient at causing a variety of diseases ranging from minor, largely self-limited skin infections to life-threatening, systemic illness and indolent, chronic infections which complicates vaccine development. We previously evaluated the ability of a selected vaccine antigen to protect against these three divergent types of *S*. *aureus* infection in mouse models of disease; the antigen exhibited varying degrees of protection against these *S aureus* disease manifestations, ranging from excellent protection to no protection, even though the infecting isolate was the same [3]. The potential reason for the lack of broad protection may, in part, stem from different growth states of the bacteria associated with different disease types, with planktonic, free-floating cocci associated with acute infections and a biofilm mode of growth associated with chronic infections. Host immune responses that are effective in containment and ultimate clearance of *S*. *aureus* infections manifested by one growth type might not be effective against another growth type.

In order to gain a better understanding of the host immune responses to different *S*. *aureus* disease types and as a first step in assessing what role those responses may play in clearance of the organism, we examined the host response to chronic, indolent infection caused by *S*. *aureus* in a biofilm and compared it to the response to an acute, self-limited *S*. *aureus* infection caused by planktonic bacteria. This comparison utilized the same bacterial isolate and the same mouse strain in order to account for potential differences attributable to responses skewed by these factors. Using these murine models in combination with immune protein arrays, we demonstrated how the responses to a chronic infection may differ from those to acute infection, as well as how the host response changes over time during biofilm infection. These data may help inform therapeutic and vaccine design.

## Materials and methods

### Ethics statement

For all animal studies, protocols were reviewed and approved by the Institutional Animal Care and Use Committees (IACUC) of the Center for Biologics Evaluation and Research (Silver Spring, MD; permit numbers 2015–03 and 2014–11). All surgeries were performed under ketamine/xylazine or isofluorane anesthesia, and all efforts were made to minimize animal suffering. Animals were sacrificed at the time points indicated below using CO_2_ inhalation.

### Strains, mice, and reagents

*S*. *aureus* USA100 strain MRSA-M2 was used to infect mice. This strain is a clinical isolate obtained from an osteomyelitis patient at the University of Texas Medical Branch [4] and was kindly provided by Mark Shirtliff at the University of Maryland School of Dentistry (Baltimore, MD). Ten-week-old female C57BL/6 mice were obtained from NCI (Frederick, MD). All animal experiments were approved by the CBER Institutional Animal Care and Use Committee (protocols 2015–03 and 2014–11), and were carried out in accordance with the recommendations in the Guide for the Care and Use of Laboratory Animals of the National Institutes of Health. Mice were fed RHI5P76 IsoPro Irradiated mouse chow, were provided water ad libitum, and were maintained on a 12 hour/12 hour light/dark cycle. Animals were monitored daily for signs of adverse clinical symptoms. Unless indicated, all reagents were obtained from ThermoFisher Scientific (Rockville, MD).

### Construction of a red fluorescent derivative of NRS384

DNA sequences flanking pseudogene USA300HOU_1102 [5, 6] were amplified by PCR using primers RP383 with RP384 and RP385 with RP386, using NRS384 chromosomal DNA as template (S1 Table). These PCR products were digested with BsaI and ligated with pMAD [7] derivative pRP1276 [8] that had been digested with BamHI and SalI. Transformation into *E*. *coli* DH5alpha and plating in the presence of ampicillin led to the isolation of plasmid pRP1302, in which the homology to the NRS384 chromosome flanks an EagI site. Separately, primers RP429 and RP433 were used to amplify TurboRFP [9] from the *B*. *anthracis* shuttle vector pRP1028 [10]. The resulting PCR product was digested with BsaI and cloned into pMAD that had been digested with NgoMIV and HindIII (removing *bgaB*), generating pRP1208. The *clpB* promoter from pMAD was amplified using primers RP426 and RP435, and the resulting PCR product was digested with BsaI and cloned into pRP1208 that had been digested with NgoMIV, generating pRP1212, in which expression of TurboRFP is under control of the *clpB* promoter. The promoter and red fluorescence gene were PCR-amplified using primers SS2228 and RP535. The resulting PCR product was digested with BsaI and cloned into pRP1302 that had been digested with EagI, yielding pRP1312, in which TurboRFP under control of the *clpB* promoter is flanked by NRS384 chromosomal homology. This plasmid was transformed into *S*. *aureus* RN4220 by electroporation [11], transduced into NRS384 using phage phi80, integrated into the NRS384 chromosome and then crossed out of the chromosome using methods described previously [6, 12]. A fluorescent and chloramphenicol-sensitive strain was isolated and was designated SAP396. Insertion of TurboRFP in the desired location was confirmed by PCR and sequencing.

### In vitro biofilm growth and confocal microscopy

Sterile 1×1 cm pieces of surgical mesh (Tyco Healthcare, Norwalk, CT) were treated with 50 μg/ml rat tail collagen in 20 mM acetic acid (Life Technologies, Grand Island, NY) for two hours at room temperature and then rinsed three times with PBS. The mesh pieces were then placed in 20 ml of Tryptic Soy Broth (TSB; Hardy Diagnostics, Santa Maria, CA) that was inoculated with an overnight culture of RFP-expressing *S*. *aureus* SAP396 at a 1:100 dilution. The mesh was incubated at 37°C for seven days, with media replacement every two days. On the seventh day, the mesh pieces were treated with 2% paraformaldehyde (Electron Microscopy Sciences, Hatfield, PA) for 15 minutes at room temperature and then rinsed in PBS followed by counter staining with Hoechst 33258. Mesh samples were then placed on Nunc 4 well chambered coverglass (ThermoFisher, Waltham, MA) and examined by SP8 DMI6000 confocal microscope (Leica, Mannheim, Germany) using a 40x objective lens (NA 1.3). Excitation wavelengths of 405 and 561 nm were used for DAPI and RFP channels respectively. Optical sections of fluorescence images were acquired and stored as lif and TIFF files for further analysis. Three dimensional reconstructed images were created by Imaris 8.4 software (Bitplane USA, Concord MA).

### Subcutaneous mesh infection

Four days prior to infection, mice were anesthetized with isoflourane and 3×3 cm areas of their backs were subjected to depilation with hair removal cream (Nair). Two days prior to infection, TSB was inoculated with a single hemolytic colony of MRSA-M2 and incubated overnight at 37°C with shaking (250 rpm). The next day, the overnight culture was diluted 1:100 in fresh TSB in a sterile petri dish. Sterile mesh sections were added and the culture was placed at 37°C with gentle shaking (40 rpm) for 24 hours. On the day of infection, the culture was decanted from the mesh, and the mesh pieces were washed three times by the addition and decantation of phosphate-buffered saline (PBS). Mice were anesthetized using 2 mg ketamine (Ketaject, Phoenix Pharmaceutical, St. Joseph, MO) and 0.1 mg xylazine (AnaSed, Akorn, Decatur, IL) and their backs were aseptically disinfected with chlorhexidine and ethanol. A small incision (approximately 1 cm) was made in the back and a single piece of either infected or sterile mesh was placed subcutaneously. The incision was closed with two to three wound clips and mice received buprenorphine XL for pain management. Several inoculated mesh pieces were retained, homogenized, and plated to determine input inocula. Mice were monitored daily for signs of incision opening or mesh migration. At designated time points, mice were euthanized using CO_2_ inhalation and mesh were aseptically removed, placed in sterile PBS, homogenized, and plated for CFU counts. For protein array experiments, excised mesh pieces were placed in sterile tissue lysis buffer (5 mM Tris-HCl pH 8, 150 mM NaCL, 1% NP-40) supplemented with a 1:100 dilution of HALT^®^ protease inhibitor cocktail, homogenized, and centrifuged at 14000 × *g* for 10 minutes. Supernatants were used for subsequent studies.

### Vancomycin treatment of subcutaneous mesh infection

Mice were implanted with MRSA-M2-inoculated mesh, or sterile mesh as controls, as described above. At day 21 post-surgery, mice implanted with MRSA-M2-inoculated mesh were split into two groups. One group was treated twice daily with subcutaneous injections of vancomycin (APP, Lake Zurich, IL) at doses of 50mg/kg while the other group received no treatment. At day 31 post-surgery, mice were euthanized and mesh were removed and homogenized for CFU counts as described above.

### Epicutaneous *S*. *aureus* infection

The epicutaneous *S*. *aureus* challenge was performed essentially as described by Prabhakara et al. [8] and Brady et al. [13]. Briefly, TSB was inoculated 1:50 with an overnight culture of MRSA-M2, and then grown at 37°C with shaking until an absorbance at 600 nm (A_600_) of approximately 0.8 was reached. The culture was then centrifuged at 5000 × *g* for 15 minutes and the pellet was resuspended in sterile PBS. The bacteria were then counted using a Petroff Hauser cell counter (Hausser Scientific, Horsham, PA). The bacteria were centrifuged again and then resuspended in PBS to a density of 1x10^11^ CFU/mL. Mice were anesthetized using isofluorane and the left ears were cleaned with 70% ethanol. The left ears were then pricked 10 times with a Morrow Brown allergy test needle (Morrow Brown Allergy Diagnostics, Oakhurst, NJ) containing a 10 μL drop of the *S*. *aureus* suspension. For controls, animals were pricked 10 times in the left ear with sterile PBS. After seven days mice were euthanized and ears were excised with scissors, placed in sterile tissue lysis buffer supplemented with a 1:100 dilution of HALT^®^ protease inhibitor cocktail, homogenized, and centrifuged at 14000 × *g* for 10 minutes. The supernatants were used for protein isolation. Alternatively, the ears were placed in sterile PBS, homogenized, and plated for CFU counts.

### Protein arrays

Explanted mesh and excised ears were homogenized in tissue lysis buffer as described above, and total protein content of supernatants was determined by bicinchoninic acid assay. Proteome Profiler Mouse XL Cytokine Arrays (R&D Systems, Minneapolis, MN) were performed per the manufacturer’s instructions. For each array, supernatants from three individual animals were pooled, and three arrays were performed per condition. Infected and control lysate arrays were run concurrently for comparison. Control samples were comprised of sterile implanted mesh for biofilm infection comparisons, and sham infected ears for SSTI comparisons. Densities were measured using ImageJ, with a fixed circular area placed over the grid-identified location for each cytokine and chemokine. Background was measured outside of the spot grid using the same fixed circular area. The limit of quantitation (LOQ) was defined as the lightest visible spot. Cytokines and chemokines that had no visible signal were assigned a density of ½ the background-subtracted LOQ (BSLOQ) value. The log_2_ fold change (LFC) of background-subtracted spot density (infected/control) was determined for each replicate for SSTI, seven-day biofilm infection, and 21-day biofilm infection. To account for variability between samples in our determination of potentially significant differences between infected and control tissue, we set stringent rules that both had to be met: The LFC of the infected:uninfected ratio had to be ≥ 1 for increased production or ≤ -1 for decreased production and the *p* value for the LFC had to be ≤ 0.05 as described below.

### Statistical analysis

All statistical analyses were performed using GraphPad Prism Version 6. For all mesh infection experiments, differences in CFU levels were evaluated using unpaired Student’s *t* test. For protein array experiments, the mean LFC for each cytokine was evaluated by for significance by one-sample *t* test, comparing the mean LFC to a theoretical mean of zero (signifying no change). To reach an alpha of 0.05, the lower bound of the 95% confidence interval must be greater than zero (for cytokines with increased production), or the higher bound must be less than zero (for cytokines with decreased production).

## Results and discussion

A fuller understanding of the host response caused by the broad range of *S*. *aureus* infection types may facilitate better design of effective vaccines and therapeutics against this pathogen. In this study, we provide a first step towards this goal by comparing the host immune response to acute and chronic *S*. *aureus* infections through use of protein arrays that semi-quantitatively measure levels of 110 murine cytokines and chemokines from host tissue. We used two models that recapitulate human disease: a self-limited skin and soft tissue infection (SSTI) model, caused by epicutaneous inoculation of *S*. *aureus* into the skin, that resolves by 14 days post-challenge [3, 8], and a surgical mesh implant model which we establish has the key characteristics of a biofilm infection.

### Mice implanted with *S*. *aureus*-inoculated surgical mesh develop chronic infection caused by a biofilm

In order to establish that the surgical mesh infection model that we used in this study represents an infection that is in fact caused by bacteria in a biofilm mode of growth, we evaluated the characteristics of the infection to verify its biofilm qualities. Hallmarks of a *S*. *aureus* biofilm infection include 1) a chronic, persistent nature; 2) bacterial cells attached to an interface or each other embedded in an extracellular polymeric matrix; and 3) resistance to antibiotics [14, 15]. The model used in our study is one initially presented by Engelsman *et al*. [16] and compared to other traditional murine models of chronic infection by Walton and colleagues [17]. Those investigators demonstrated that the surgical mesh model provided long-standing infections with durations up to 10 and 35 days, respectively. However, neither study evaluated fully whether the *S*. *aureus* associated with the mesh implants were growing as a biofilm. Therefore, as part of our adaptation of this model, we undertook several steps to characterize the infection model more completely.

**Fig 1:**
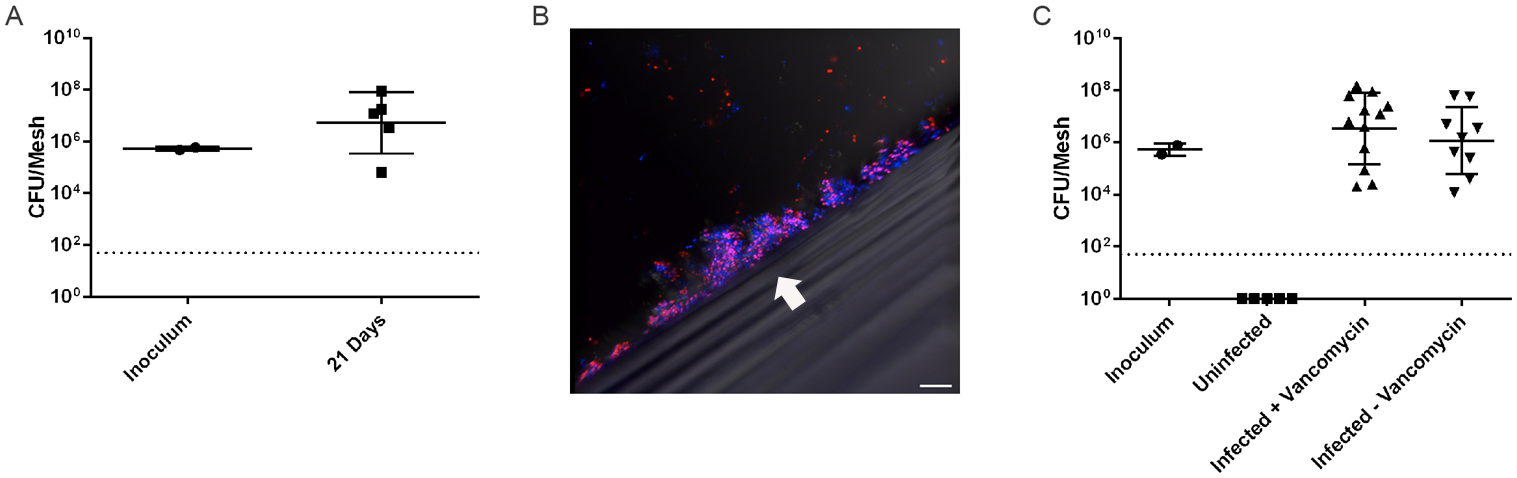
Characterization of *S*. *aureus* surgical mesh model of biofilm infection. **(A)** Surgical mesh sections were incubated for 24 hours in *S*. *aureus* liquid culture before implantation. Shown are mesh implants removed 21 days after implantation compared to CFU levels obtained immediately before implantation (inoculum). Individual implants with mean +/- SD are shown. **(B)** 1 x 1 cm segments of sterile surgical mesh were incubated in TSB containing RFP-expressing *S*. *aureus* for 7 days. A 3D reconstructed confocal microscopy image from 28 optical sections overlaid with differential interference contrast (DIC) image is shown. RFP-expressing *S*. *aureus* are red and extracellular DNA is blue (Hoescht 33258). The mesh interface is indicated with the arrow. Scale bar indicates 10 μm. **(C)** Inoculated or sterile mesh were implanted, and 21 days after implantation, animals were either treated with vancomycin (infected + vancomycin) or left untreated (infected–vancomycin). CFU levels for individual implants +/- SD are shown.

For our biofilm infection studies, we used *S*. *aureus* isolate MRSA-M2, which is a clinical isolate obtained from an osteomyelitis patient and has been used in other *in vivo* biofilm infection models [4, 18–20], and C57BL/6 mice, which are expected to manifest infections similar to those seen in humans [20]. We followed the mice for 21 days, after which we euthanized the animals and removed the mesh. The mesh pieces were coated in host tissues and most infected mesh pieces had similar physical characteristics to control mesh (i.e., no pus or abscess formation at the implant site), suggesting that the infection was not acute. *S*. *aureus*-inoculated mesh remained colonized at 21 days with *S*. *aureus* at levels similar to those seen after initial inoculation (Fig 1a). The lack of acute symptoms or infection spread, as well as persistent colonization in immunocompetent mice suggests that the mesh infection is chronic in nature and persists in the face of the host immune response, confirming previous findings [16, 17].

To demonstrate that *S*. *aureus* grows on the mesh in a three-dimensional structure surrounded by extracellular matrix material, we utilized SAP396, an isolate of *S*. *aureus* expressing red fluorescent protein (RFP), which could be readily visualized by confocal microscopy. After seven days of growth in vitro, mesh incubated with SAP396 exhibited colonization by the bacteria, which occurred in three dimensional structures on the mesh surface (Fig 1b). Hoeschst 33258 staining for DNA showed substantial amounts of DNA surrounding the bacteria. This DNA is likely extracellular DNA released from lysed staphylococci [21]. Control mesh that was incubated in sterile media did not have any bacteria or extracellular DNA visible on its surface (data not shown).

Finally, to further verify the biofilm nature of the infection, we examined susceptibility of the bacteria to killing by antibiotics [22]. Because MRSA-M2 is susceptible in its planktonic form to vancomycin, we performed our mesh infection model as described above, but added a ten day course of vancomycin treatment on day 21 post-implantation. Mice were administered 50 mg of vancomycin/kg twice daily for ten days. On day 31, mice were euthanized and mesh were harvested and plated for CFU levels. Control animals, which received *S*. *aureus*-inoculated implants but did not undergo vancomycin treatment, remained infected to the same level seen at day 21. Animals that received *S*. *aureus* inoculated implants and vancomycin treatment demonstrated CFU levels that were not significantly different from those of the animals that did not receive vancomycin (Fig 1c). We tested the ability of vancomycin to inhibit MRSA-M2 growth during planktonic culture and confirmed the MIC to be between 0.5 and 2 μg/ml (data not shown).

Overall, our combined data demonstrating three-dimensional biofilm structure in vitro and prolonged infections in an immunocompetent host, both with and without antibiotic pressure, provides strong evidence that this mesh model accurately represents a chronic *S*. *aureus* biofilm infection.

### Comparison of the host immune response to acute SSTI and chronic surgical mesh infection

Most planktonic *S*. *aureus* infections are cleared by the host whereas biofilm infections are often chronic in nature. While differences in bacterial growth may have an effect, differences in the host immune response to acute versus chronic infections may also play a role. Some studies have begun to attempt to dissect the host response to *S*. *aureus* biofilm infection [19, 20, 23]. However, a direct, simultaneous comparison between acute, self-limited *S*. *aureus* infection where the immune system functions to eradicate the infection, and chronic biofilm infection where the response is incapable of clearance, is currently lacking.

We chose to directly compare murine cytokine and chemokine profiles during these two infection types, using the same mouse strain and the same *S*. *aureus* isolate for each model. To this end, we utilized the SSTI model developed previously by Prabhakara *et al*. [8] to represent an acute infection caused by planktonic *S*. *aureus* and the surgical mesh model described above which represents chronic biofilm infection. For the SSTI model, we infected C57BL/6 mice in the ear pinnae with MRSA-M2. Mesh biofilm infections were carried out as described above. We considered the kinetics of response in both models when choosing time points for evaluation. We harvested SSTI tissue at day 7 post-infection, which is the peak of disease [8], and which had a similar gene expression profile to earlier time points of infection in our previous RNA-seq study [13]. We also harvested implant-associated tissue at this time, which would represent an early biofilm infection in which the biofilm is not yet fully established but the infection is not characterized by a high degree of planktonic bacteria. Additionally, other *S*. *aureus* biofilm infection models suggest that at day 7 tissue damage becomes apparent [24], CFU levels stabilize [19], and biofilm mass is first detected [25], but the bacteria are still metabolically active [24, 25]. We also evaluated the host response in the biofilm infection model at 21 days post-implantation, which would represent chronic disease. We evaluated cytokine levels in infected samples in comparison to those from mock-infected samples to account for any changes due to surgery or wounding. Tissue lysates were incubated with Mouse XL Cytokine Arrays and background-adjusted spot densities were compared as described in Materials and Methods. A list of all proteins that met these thresholds for at least one infection type is provided in Table 1. The complete data set for all proteins is provided in S2 Table.

**Table 1:** Proteins with significant differences in acute and/or chronic *S*. *aureus* infection.

	SSTI			Day 7 Mesh			Day 21 Mesh		
	Mean LFC^a^	95% CI	p value	Mean LFC	95% CI	p value	Mean LFC	95% CI	p value
BAFF	***3*.*8***	***0*.*51 to 7*.*1***	***0*.*038***	***2*.*2***	***0*.*37 to 4*.*0***	***0*.*035***	0.69	-0.087 to 1.5	0.062
C1qR1	***1*.*1***	***0*.*33 to 1*.*9***	***0*.*026***	-0.017	-2.7 to 2.6	0.981	-0.75	-1.4 to -0.15	0.033
CCL17	0.69	-2.6 to 4.0	0.462	-2	-5.0 to 0.93	0.098	***-1*.*8***	***-3*.*0 to -0*.*55***	***0*.*025***
CD14	2.8	-0.51 to 6.2	0.068	***4*.*4***	***2*.*9 to 5*.*9***	***0*.*006***	1.7	-4.1 to 7.5	0.331
CD40	***1*.*6***	***0*.*33 to 2*.*8***	***0*.*032***	-0.65	-4.3 to 3.0	0.523	-0.23	-1.1 to 0.60	0.352
CD160	-0.11	-5.3 to 5.1	0.935	***-2*.*3***	***-2*.*3 to -2*.*3***	***< 0*.*001***	-0.12	-5.9 to 5.6	0.938
C5/C5a	1.5	-5.2 to 8.3	0.426	***3*.*5***	***1*.*4 to 5*.*6***	***0*.*019***	2.2	-3.2 to 7.6	0.224
Complement factor D	***1*.*5***	***0*.*97 to 2*.*1***	***0*.*007***	0.004	-1.6 to 1.6	0.993	-0.34	-1.8 to 1.1	0.426
CRP	***2*.*2***	***0*.*50 to 4*.*0***	***0*.*031***	0.6	-1.3 to 2.5	0.305	0.041	-0.37 to 0.46	0.712
CXCL1	3.1	-0.33 to 6.4	0.060	***3*.*5***	***1*.*1 to 6*.*0***	***0*.*026***	2.1	-3.1 to 7.3	0.228
CXCL2	***5*.*4***	***3*.*4 to 7*.*5***	***0*.*008***	***6*.*7***	***2*.*8 to 11***	***0*.*017***	4.5	-1.0 to 10	0.072
CXCL9	***4*.*9***	***0*.*42 to 9*.*4***	***0*.*042***	0.73	-4.2 to 5.7	0.591	***2***	***0*.*33 to 3*.*6***	***0*.*036***
CXCL10	***1*.*8***	***0*.*058 to 3*.*4***	***0*.*047***	-0.024	-1.7 to 1.7	0.958	-0.17	-0.83 to 0.49	0.385
Flt-1 ligant	***-1*.*3***	***-2*.*5 to -0*.*02***	***0*.*049***	-0.024	-1.7 to 1.7	0.958	0.19	-1.3 to 1.7	0.650
HGF	2.1	-0.81 to 5.0	0.090	***1*.*3***	***0*.*66 to 2*.*0***	***0*.*013***	0.7	-0.31 to 1.7	0.097
IGFBP-6	-0.22	-0.88 to 0.43	0.283	***-1*.*2***	***-2*.*3 to -0*.*19***	***0*.*037***	-0.084	-1.5 to 1.4	0.828
IL-1a/IL-1F1	-0.061	-0.65 to 0.52	0.696	***7*.*9***	***6*.*4 to 9*.*3***	***0*.*002***	5.6	-0.52 to 12	0.059
IL12P40	***1*.*2***	***0*.*47 to 1*.*9***	***0*.*019***	-0.024	-1.7 to 1.7	0.958	-0.6	-3.2 to 2.0	0.420
IL17A	-0.061	-2.2 to 2.1	0.912	***4*.*7***	***0*.*77 to 8*.*7***	***0*.*036***	4	-5.3 to 13	0.206
LIX (CXCL5)	***5*.*4***	***2*.*9 to 7*.*8***	***0*.*011***	0.37	-1.1 to 1.8	0.386	0.61	-0.43 to 1.6	0.127
MMP-9	***5*.*4***	***1*.*5 to 9*.*3***	***0*.*027***	0.22	-0.13 to 0.56	0.113	0.59	-0.59 to 1.8	0.163
Pentraxin 3	***1*.*3***	***0*.*095 to 2*.*6***	***0*.*044***	-1.5	-5.7 to 2.6	0.250	0.51	-2.2 to 3.2	0.503
Pref-1	0.55	-1.9 to 3.0	0.444	**-2.7**	**-4.1 to -1.2**	**0.015**	-0.52	-4.4 to 3.4	0.619

Bold font indicates that the LFC met our significance criteria of a mean LFC ≥1 or ≤-1 and a *p* value ≤ 0.05.

^a^Mean LFC: The Log_2_ ratio of background-subtracted spot densitometry for infected compared to uninfected tissue lysate.

**Fig 2:**
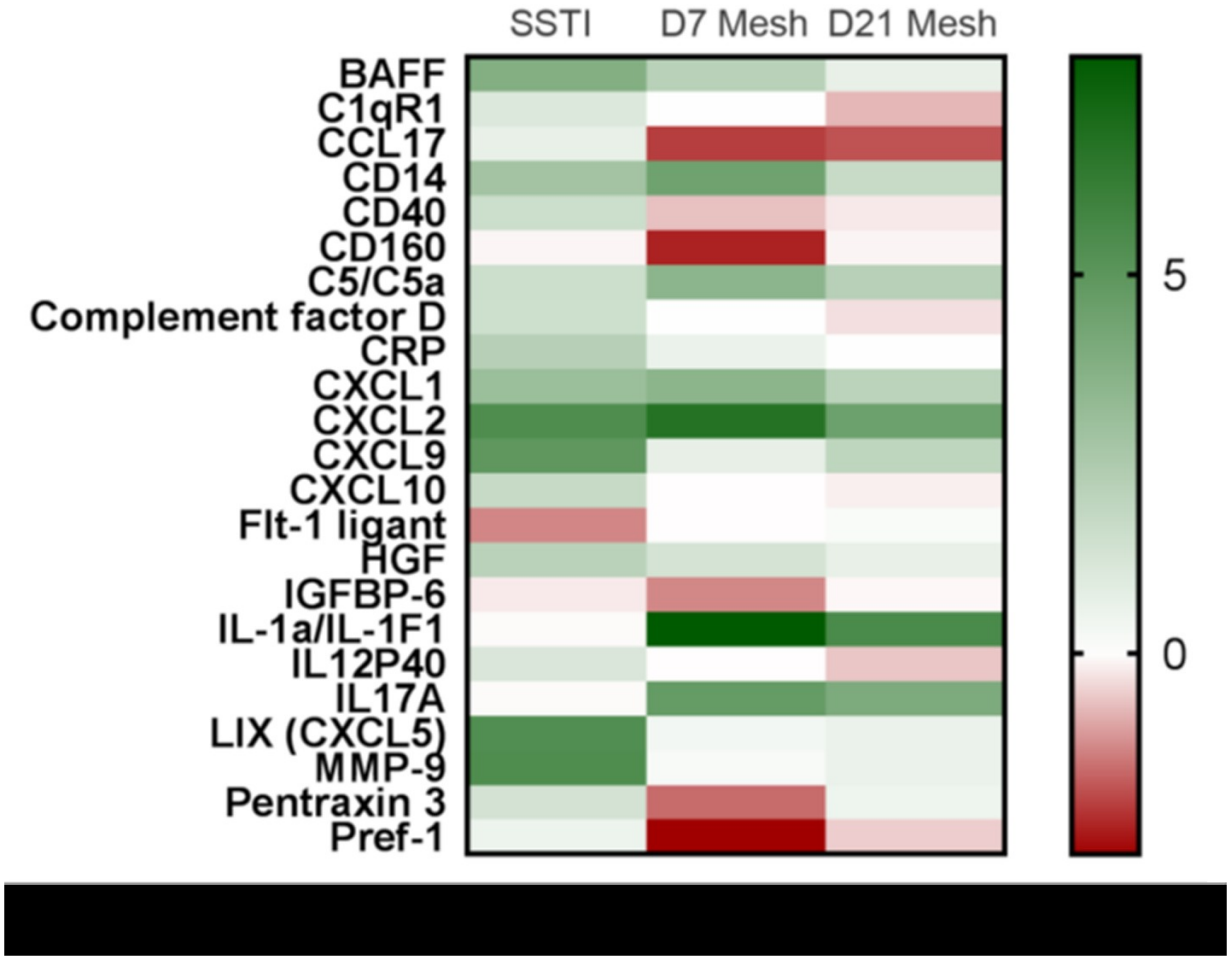
Protein production levels in acute and chronic *S*. *aureus* infection. Data from three independent arrays, each run with pools of three animals, were analyzed. Only proteins that met significance criteria (Log2 fold change (LFC, infected:control) ≤ -1 or ≥ 1, and p ≤ 0.05, one sample t test) in at least one infection type are shown. LFC is represented by the range indicated on the right, with proteins with increased production indicated with green and proteins with decreased production indicated with red. No change relative to control (uninfected) samples is indicated in white.

Out of 110 cytokines and chemokines on the array, 13 met our significance criteria in SSTI, 11 met the criteria at day 7 during mesh biofilm infection, and only two met the criteria at day 21 during mesh biofilm infection. Heat map analysis illustrates that production trends patterned differently between acute SSTI and chronic biofilm infections; proteins that were significantly affected in SSTI tended to not be affected during biofilm infection, and proteins that were significantly altered during biofilm infection were not affected during SSTI (Fig 2). The response during late biofilm infection (day 21) was decreased compared to that during early biofilm infection (day 7), though the overall pattern of what proteins were affected was similar.

We noted significantly increased levels of certain CXC chemokines, particularly in SSTI and early in the biofilm infection. The CXC chemokines are chemotactic for neutrophils, which have been demonstrated as important for resolution of SSTI [26]. CXCL9 is significantly increased in the 21 day biofilm infection as well as during SSTI. In addition, CXCL10 is also significantly increased in SSTI. These two chemokines are important for recruitment of CXCR3-expressing Th1 cells [27, 28]. The increase in these chemokines, as well as the rise in IL-12p40 levels, is suggestive of a TH1 response at day 7 during SSTI, which was also suggested in previous work using RNA-seq analysis [13]. The significant increase of CXCL9 during a chronic infection suggests that the contribution of this chemokine is ineffective at clearing the biofilm. CXCL2 was significantly increased during SSTI, and at our day 7 biofilm infection time point. The level of CXCL2 in our model is opposite that in the catheter model used by Thurlow *et al*., where ELISA of infected catheter tissue showed a significant decrease in CXCL2 production. While Thurlow and colleagues also found significantly decreased CCL2 and IL-1β levels in infected catheter tissue, the levels of these cytokines did not differ between infected and uninfected control mesh in our mesh model [23]. On the other hand, the increased levels of *CXCL1 and* CXCL2, as well as the increased production of IL-1α and IL17-a, suggest that the mesh biofilm model promotes an early TH1 and/or TH17 response. These data correlate with what Prabhakara *et al*. noted with their tibial implant model [19, 20]. CXCL1 and CXCL2 are critical for neutrophil recruitment [29]. Future investigations into the question of why these responses are ineffective in reducing bacterial loads in *S*. *aureus* biofilm infections would be of interest.

Comparison of significantly altered protein levels during early (Day 7) versus late (Day 21) biofilm infection suggests that the response is similar over time; however, the response level is decreased by 21 days compared to day 7 post-implantation. These results suggest that the overall response does not change, but simply decreases as the infection progresses to a chronic state. The bacteria may be blunting the immune response during this transition from early to late biofilm infection, as was suggested by Thurlow et al. [23]. However, we do not see evidence of this in our data. By three weeks of biofilm infection, *S*. *aureus* has reached a mature state, which involves a transition by the majority of biofilm-associated bacteria into dormancy [30]. This transition likely means that fewer virulence factors or pro-inflammatory mediators are present, which may lead the host response to decrease. *S*. *aureus* may also be evading the host response by invading host cells, including professional and non-professional phagocytes [31]. Future studies evaluating staphylococcal factors that are up- or down-regulated as biofilm infection progresses in this model could help elucidate what bacterial factors may play a role in the dampened response during chronic infection.

In both infection models, we note cytokine and chemokine responses that are suggestive of inflammatory responses at the infection site, though the specific factors produced in each infection somewhat differ. LIX/CXCL5 and MMP-9 are both produced at a higher level in infected skin relative to controls than in infected mesh at either early or late time points. These factors are both associated with neutrophils; MMP9 is found stored in tertiary granules and plays a role in activating several inflammatory cytokines [32], while CXCL5 is chemotactic for neutrophils and was shown previously to be induced during SSTI [26]. These data imply that there may be a higher influx of neutrophils to the site of SSTI compared to biofilm infection. However, we do detect high levels of both CXCL2 and IL-17a in biofilm-associated tissue at the same seven day time point during biofilm infection. Pref-1 (DLK1) levels are significantly lower in infected mesh implants at day 7 relative to controls. This factor is postulated to inhibit pro-inflammatory responses [33], so its decreased production may support an increase in inflammation at the site of mesh infection. Perhaps the level of inflammation induced by biofilm infection is sufficient for providing a nutrient niche for *S*. *aureus* [20], but insufficient for clearing the infection. We also note significantly decreased production of CD160 and IGFBP-6 during early biofilm infection. CD160 is a receptor found on NK cells and T lymphocytes [34], and IGFBP-6 has been shown to be present on T lymphocytes and to induce chemotaxis [35], so their decreased levels in biofilm may be evidence of decreased T cell homing to the site of infection.

Overall, the data suggest that the immune response to *S*. *aureus* biofilm infection is attenuated compared to the response to acute SSTI, particularly as the biofilm infection progresses to a chronic, persistent disease state. It is likely that there are several nuanced systems at play in responses to both disease manifestations, and further work is needed in order to begin to test the contributions of individual immune factors in both SSTI and biofilm infection. To our knowledge, this work is the first to present a simultaneous comparison of the host response to acute and chronic *S*. *aureus* infection, where potential confounding factors such as mouse strain and infecting isolate are controlled. Further work is needed in order to begin to elucidate the contributions of individual immune factors in both SSTI and biofilm infection.

## Conclusion

Using mouse models of acute SSTI and chronic mesh implant infection, we compared and contrasted the immune response during *S*. *aureus* infections caused by bacteria in planktonic and biofilm modes of growth. We also characterized the mesh model of *S*. *aureus* infection in order to demonstrate that the model results in a chronic biofilm that is resistant to clearance both by the host immune system and antibiotic treatment. Through use of proteome array analysis comparing SSTI to mesh infection, we were able to curate a descriptive list of cytokines and chemokines that are significantly changed in their production levels, compared to uninfected controls, during both acute and chronic *S*. *aureus* infection. We found that more cytokines and chemokines are significantly altered during SSTI than during late biofilm infection. We also found that over time, the magnitude, but not the overall pattern of cytokine expression, changes during biofilm infection. Our results suggest that the biofilm infection may promote a pro-inflammatory TH17 environment while down-regulating chemokines important to T cell homing to the site of infection. Further investigation into the contribution of the specific cytokines and chemokines that are differentially produced during self-limited acute infections compared to during chronic biofilm infection may be useful in beginning to differentiate an effective anti-*S*. *aureus* immune response. This information may support the design of novel therapeutic and vaccination strategies against *S*. *aureus* biofilm infection.

## Supporting information

S1 Table Primer sequences for construction of RFP-expressing *S*. *aureus* strain SAP396. (XLS)

S2 Table Complete data set for Mouse Xl Cytokine Arrays. The mean LFC, upper and lower bounds of the 95% confidence interval and p value are presented for each arrayed cytokine and chemokine for day 7 SSTI, day 7 mesh infection, and day 21 mesh infection. Those proteins meeting our significance criteria (outlined in Materials and methods) are highlighted in bolded italics.
(XLSX)
